# Shortening surgical training through robotics: randomized clinical trial of laparoscopic *versus* robotic surgical learning curves

**DOI:** 10.1002/bjs5.50353

**Published:** 2020-10-14

**Authors:** T. M. H. Gall, W. Alrawashdeh, N. Soomro, S. White, L. R. Jiao

**Affiliations:** ^1^ The Academic Surgical Unit The Royal Marsden Hospital, Imperial College London UK; ^2^ Hepatopancreatobiliary Surgical Unit Newcastle upon Tyne UK; ^3^ Department of Urology The Freeman Hospital Newcastle upon Tyne UK

## Abstract

**Background:**

Minimally invasive surgery is the standard technique for many operations. Laparoscopic training has a long learning curve. Robotic solutions may shorten the training pathway. The aim of this study was to compare laparoscopic with robotic training in surgical trainees and medical students.

**Methods:**

Surgical trainees (ST group) were randomized to receive 6 h of robotic or laparoscopic simulation training. They then performed three surgical tasks in cadaveric specimens. Medical students (MS group) had 2 h of robotic or laparoscopic simulation training followed by one surgical task. The Global Rating Scale (GRS) score (maximum 30), number of suture errors, and time to complete each procedure were recorded.

**Results:**

The median GRS score for the ST group was better for each procedure after robotic training compared with laparoscopic training (total GRS score: 27·00 (i.q.r. 22·25–28·33) *versus* 18·00 (16·50–19·04) respectively, *P* < 0·001; 10 participants in each arm). The ST group made fewer errors in robotic than in laparoscopic tasks, for both continuous (7·00 (4·75–9·63) *versus* 22·25 (20·75–25·25); *P* < 0·001) and interrupted (8·25 (6·38–10·13) *versus* 29·50 (23·75–31·50); *P* < 0·001) sutures. For the MS group, the robotic group completed 8·67 interrupted sutures with 15·50 errors in 40 min, compared with only 3·50 sutures with 40·00 errors in the laparoscopic group (*P* < 0·001) (10 participants in each arm). Fatigue and physical comfort levels were better after robotic compared with laparoscopic operating for both groups (*P* < 0·001).

**Conclusion:**

The acquisition of surgical skills in surgical trainees and the surgically naive takes less time with a robotic compared with a laparoscopic platform.

## Graphical abstract

Medical students and surgical trainees were randomized to robotic or laparoscopic simulator training followed by cadaveric surgical tasks. The robotic groups had a shorter learning curve with better task performance.

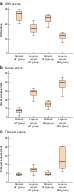

Another advantage for robotic surgery

## Abstract (es)

**Antecedentes:**

La cirugía mínimamente invasiva (*minimally invasive surgery*, MIS) es la técnica considerada como el patrón oro para muchas intervenciones quirúrgicas. La formación en laparoscopia tiene una curva de aprendizaje larga. El empleo de la robótica puede acortar dicho periodo de entrenamiento. El objetivo de este estudio fue comparar el entrenamiento laparoscópico con el robótico en residentes de cirugía y estudiantes de medicina.

**Métodos:**

Los integrantes del grupo de residentes de cirugía (*surgical trainees*, ST) fueron aleatorizados a recibir 6 horas de entrenamiento con simulación laparoscópica o robótica. Seguidamente, efectuaron tres procedimientos quirúrgicos en especímenes de cadáver. El grupo de estudiantes de medicina (*medical students*, MS) recibió 2 horas de entrenamiento de simulación laparoscópica o robótica, seguidas de un procedimiento quirúrgico. Se registró el resultado de la escala global de puntuación (*global rating scale score*, GRS, máxima puntuación = 30), el número de errores cometidos en las suturas y el tiempo para completar cada procedimiento.

**Resultados:**

La mediana de GRS para el grupo ST fue mejor para cada procedimiento tras el entrenamiento robótico (GRS puntuación total 27,00 ± 6, *n* = 10) en comparación con el entrenamiento laparoscópico (18,00 ± 5, *n* = 10, *P* < 0,001). Se cometieron menos errores en las tareas robóticas en comparación con las laparoscópicas, tanto para las suturas continuas (7,00 ± 5 y 22,25 ± 5; *P* < 0,001) como discontinuas (8,25 ± 4 y 29,50 ± 8; *P* < 0,001). En el grupo de MS, el grupo de robótica completó 8,67 suturas discontinuas con 15,50 errores en 40 minutos, en comparación con solo 3,50 suturas con 40,00 errores en el grupo laparoscópico (*P* < 0,001). Los niveles de fatiga y de confort físico fueron mejores tras la cirugía robótica en comparación con la laparoscópica (*P* < 0,001) para ambos grupos.

**Conclusión:**

En los residentes de cirugía y en personas sin contacto previo con la cirugía, las habilidades quirúrgicas se adquirieron en menos tiempo con una plataforma robótica que laparoscópica.

## Introduction

The past three decades have witnessed the rapid emergence of minimally invasive surgery (MIS). The advantages of MIS over open operations include less pain, less blood loss and faster return to functional activities. Widespread adoption has been slow, however, particularly for long operations and those involving complex anastomoses in specialties such as hepatopancreatobiliary and vascular surgery. Barriers to establishing laparoscopic practice include operator discomfort and fatigue, physiological tremor that is amplified through the length of the instruments, and limited instrument motion. Laparoscopic surgery requires a significant amount of time and training before competency in basic skills is reached and, even after proficiency has been achieved, experienced surgeons may find a long learning curve for individual operations. The number of cases after which operating time and morbidity is reduced may be as high as 85 for laparoscopic colectomy^1^, 100 for laparoscopic urological procedures^2^, and over 100 for laparoscopic pancreatoduodenectomy^3^.

Robotic surgery has several advantages over the laparoscopic approach. It provides a three‐dimensional visual field with depth perception. Its ‘wristed’ instruments provide the natural seven degrees of motional freedom, mimicking open surgery. These advances increase dexterity and improve hand–eye coordination. The learning curve for robotic operations may be shorter than the conventional laparoscopic approach across surgical specialties^4^, ^5^, ^6^, ^7^, ^8^, ^9^.

The present study was designed to establish whether the acquisition of minimally invasive surgical skills, including suturing and performance of basic operations, differed between robotic and laparoscopic techniques in novice surgeons and in the surgically naive.

**Fig. 1:**
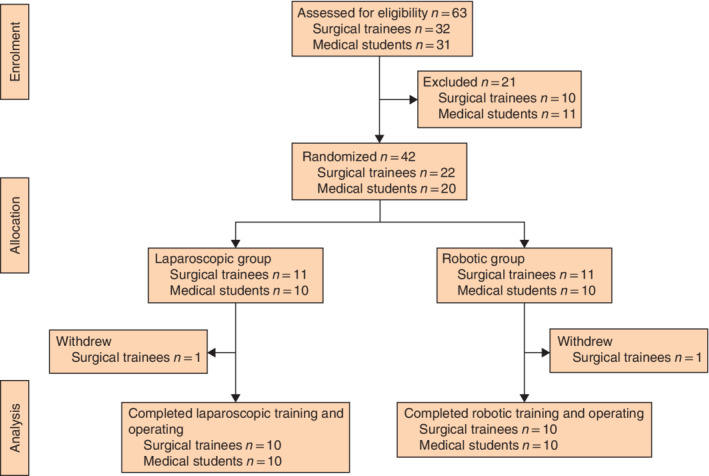
CONSORT diagram for the study

## Methods

The trial was registered retrospectively on the ISRCTN registry (study ID ISRCTN36193711).

### Design and randomization

This was a randomized parallel‐group trial investigating laparoscopic *versus* robotic training in junior surgical trainees and medical students. Surgical trainees from the North‐West Thames London Deanery and the North‐East Deanery in the UK, as well as medical students from Imperial College London and Newcastle University, were invited to participate. Participants were invited to the centre and blinded to their group until the training day. Eligible participants were computer‐randomized in a 1 : 1 ratio between laparoscopic and robotic training. Both groups received either 6 h of robotic or laparoscopic simulation and box training, followed by recorded cadaveric operating. Randomization was stratified to training deanery and level of training. The trial was conducted at the Newcastle Surgical Training Centre, Freeman Hospital, Newcastle upon Tyne, UK. This training centre is licensed to train students on human cadavers (Human Tissue Act 2004; licensing number 12148).

Inclusion criteria for surgical trainees (ST group) were: UK surgical trainee with knowledge of anatomy and steps of cholecystectomy. For medical students (MS group) the criterion was that they should attend a UK medical school and be in training years 3–5. Surgical trainees were excluded if they had more than 4 years of postgraduate training or had performed more than five laparoscopic or robotic cholecystectomies as the primary surgeon. Medical students were excluded if they had previously assisted at any MIS.

### Training

#### Surgical trainee group

Laparoscopic training involved 3 h on a laparoscopic virtual reality simulator (LAPMentor™ II; 3D Systems, Littleton, Colorado, USA) to complete laparoscopic basic skills, essential tasks and basic suturing skills, plus 3 h on a box trainer (Body Torso Trainer; Pharmabotics, Winchester, UK), where they completed 1 h of depth perception tasks, including stacking sugar cubes and peg transfer, and 2 h of suturing tasks, performing interrupted and continuous suturing on skin suture pads.

Robotic training involved 3 h on a robotic virtual reality simulator (da Vinci® Xi; Intuitive, Sunnyvale, California, USA) to completed level 1 endowrist manipulation, camera targeting and basic suturing tasks, plus 3 h on a box trainer (Body Torso Trainer), as described above.

#### Medical student group

Laparoscopic training involved 1 h of depth perception tasks on the laparoscopic box trainer and 1 h of suturing on skin suture pads.

Robotic training involved 1 h on a virtual reality robotic simulator (da Vinci® Xi) to completed endowrist manipulation tasks and 1 h of suturing tasks, as described above.

### Cadaveric operating

#### Surgical trainee group

All participants completed the cadaveric operation 1 day after the above training, either laparoscopically or robotically according to their randomized group. All procedures were timed and video‐recorded.

Ports were inserted before the start of each procedure by a laparoscopically and robotically trained general surgeon. Carbon dioxide insufflation was set to a pressure of 14 mmHg. The laparoscopic torsos had a 12‐mm subumbilical port, 5‐mm epigastric port, and two 5‐mm right upper quadrant (RUQ) ports inserted for the cholecystectomy. A 12‐mm port in the left upper quadrant and a 5‐mm RUQ port were subsequently inserted for the suturing procedures. The robotic torsos had four 8‐mm ports inserted in a horizontal line at the level of the umbilicus. A 12‐mm assistant port was inserted between, and 5 cm inferior to, arms 1 and 2 of the da Vinci® Xi system.

Elements of three operative procedures were undertaken: cholecystectomy involving either hepatocystic triangle or liver bed dissection, where the fundus of the gallbladder was retracted above the liver by an assistant (laparoscopically) or by arm 4 (robotically) by a trained surgeon before the start of the timed procedure; gastrostomy closure with continuous suture after a 5‐cm gastrostomy had been made by a trained surgeon, using 3·0 polyglactin sutures (Ethicon, Somerville, New Jersey, USA) cut to a 14‐cm length, where time to complete the procedure was started after insertion of the first suture; and small bowel end‐to‐end anastomosis with interrupted sutures, where transection of the jejunum was performed by a trained surgeon. Again, all sutures were 3·0 polyglactin (Ethicon) and cut to a 14‐cm length. Time to complete the procedure was started after insertion of the first suture and ended after either six completed interrupted sutures or at 40 min.

#### Medical student group

All participants completed the cadaveric operation on the same day as the training.

Ports were inserted as above for laparoscopic and robotic operations. Each participant performed interrupted suture closure of a previously made 5‐cm gastrostomy incision. All sutures were 3·0 polyglactin (Ethicon) and cut to a 14‐cm length. The number of completed sutures within 40 min was recorded.

### Assessment

The time taken for each procedure was recorded in real time. All procedures were recorded, and video analysis was completed subsequently and independently by two hepatobiliary surgeons. Each procedure was rated according to the previously validated Global Rating Scale (GRS) (*Table* *1*)^10^. Each suturing task was also scored by the number of errors performed in line with the Van Sickle assessment^11^ (*Table* *2*). Each assessor then rescored three robotic and three laparoscopic videos at least 4 weeks after the first assessment.

**Table 1:** Global Rating Scale scoring system

Respect for tissue	1	2	3	4	5
	Frequent unnecessary force on tissues or caused damage by inappropriate use of instruments		Careful handling of tissue but occasionally caused damage		Carefully handles tissue appropriately with minimal damage to tissues
**Time and motion**	**1**	**2**	**3**	**4**	**5**
	Many unnecessary moves		Efficient time motion, but some unnecessary moves		Clear economy of movement; maximum efficiency
**Instrument handling/knowledge**	**1**	**2**	**3**	**4**	**5**
	Tentative/awkward moves or inappropriate use		Competent use of instruments; occasionally awkward		Fluid moves with instruments; no awkwardness
**Flow of operation**	**1**	**2**	**3**	**4**	**5**
	Stopped frequently, seemed unsure of next move		Some forward planning; reasonable progression		Obviously planned course; effortless flow
**Depth perception**	**1**	**2**	**3**	**4**	**5**
	Consistently overshoots, swings wide, slow to correct		Some overshooting but quick to correct		Accurately directs instruments to correct plane
**Bimanual dexterity**	**1**	**2**	**3**	**4**	**5**
	Uses only one hand, poor coordination between hands		Uses both hands, but does not optimize their interaction		Expertly uses both hands to provide optimal exposure

**Table 2:** Van Sickle assessment^11^ of suture errors used for video assessment

Error	Description
Missed grasp	Jaws of the instrument are opened and closed without retaining the desired target (either suture, tissue or needle)
Instrument not assisting	The instrument not holding the suture is not actively engaged in assisting in the performance of the step or is out of view while not actively participating in the procedure (holding exposure, holding the suture)
Tear/injure tissue	Tearing tissue with either manipulation or retraction, a placed suture tearing through the tissue, or tissue injury that causes bleeding from contact with the needle
Excessive manipulation	Either the needle or the suture is grasped more than two times during a step. The contact of the grasper with the suture to slide the knot down does not count
Incomplete or repeated bite	Once the tip of the suture needle engages the target tissue, it is either disengaged or fails to traverse the tissue completely (the tip is not seen, or once seen does not remain visible) without additional manipulation
Needle out of view	A grasped needle is completely out of view. Grasped suture with a hanging needle does not count. If the needle is out of view due to a primary scope problem then an error is not scored
Missed loop	Once an attempt to loop the suture around the instrument is initiated, it is not completed
Tail looped	When the suture tail is pulled through to make a knot, it loops and requires a release and additional manipulation to free the loop
Failure to square knot	Once the slipknot is in place, it is not squared
Attending takeover	The attending surgeon has to demonstrate or perform any aspect of suturing or tying
Scissors touch tissue	The non‐shaft portion of the scissors touches tissue

**Fig. 2:**
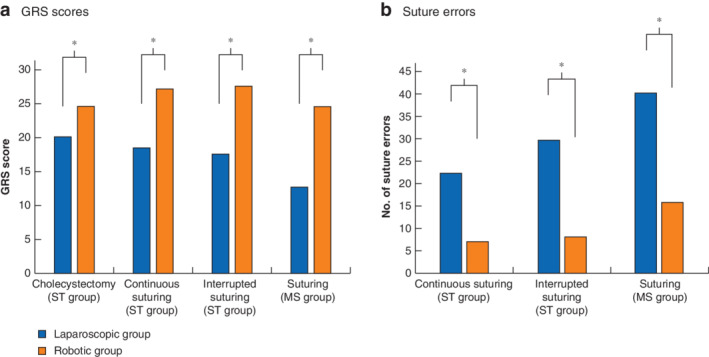
Global Rating Scale scores and suture errors in surgical trainee and medical student groups **a** Global Rating Scale (GRS) scores and **b** number of suture errors in surgical trainee (ST) and medical student (MS) groups. Values are median. *P < 0·050 (Mann–Whitney *U* test).

The primary outcome measure assessed was the GRS score for each procedure. Secondary outcomes were: time taken for each procedure; number of suturing errors for each procedure; number of loops created with continuous suture closure of gastrostomy (ST group); number of sutures completed in 40 min (MS group); surgeon comfort after all procedures; and surgeon fatigue after all procedures.

### Statistical analysis

A sample size calculation was performed based on the GRS scores for each laparoscopic and robotic procedure in a pilot of six participants. For this total of nine laparoscopic and nine robotic GRS scores, the mean(s.d.) scores were 23·7(3·61) and 18·2(5·32) for the robotic and laparoscopic group respectively. Using a significance level of 0·05 and power of 80 per cent, a total of seven participants in each group was required for the study.

Statistical analysis was undertaken with IBM SPSS® Statistics v25 (IBM, Armonk, New York, USA). Two group comparisons were made using Student's *t* test for continuous parametric data, the Mann–Whitney *U* test for continuous non‐parametric data, and Fisher's exact test for categorical data. Interassessor and intra‐assessor reliability was assessed using the interclass correlation coefficient (ICC) and intraclass correlation coefficient (IntraCC), reported with 95 per cent confidence intervals and associated *P* value. *P* < 0·050 was considered statistically significant.

## Results

A total of 63 participants were recruited, consisting of 32 surgical trainees and 31 medical students. Of these, 21 failed to meet the inclusion criteria. The final 42 participants (22 surgical trainees and 20 medical students) were randomized into either the laparoscopic or the robotic group (*Fig*. *1*). Two surgical trainees were unable to attend the training following randomization. Over a 10‐day period, 20 surgical trainees and 20 medical students completed the training and cadaveric operating.

### Surgical trainee group

Ten surgical trainees were randomized to the laparoscopic group and ten to the robotic group. Group characteristics and trainees' previous experience were comparable (*Table* *3*). Only one trainee played video games regularly for about 3 h per week. No participant had any previous robotic simulation experience or any laparoscopic or robotic intraoperative suturing experience.

**Table 3:** Baseline characteristics of surgical trainee group and outcomes of laparoscopic *versus* robotic surgical tasks

	Laparoscopic group (*n* = 10)	Robotic group (*n* = 10)	*P*
**Trainee characteristics**			
Length of training (years)*	2·20(1·23)	2·20(1·03)	1·000§
Training Deanery			1·000**‡**
Newcastle	5	4	
London	5	6	
Sex ratio (M : F)	4 : 6	3 : 7	0·639**‡**
No. of laparoscopic cholecystectomies*			
Performed as assistant	18·90(14·65)	21·80(17·13)	0·689§
Partially performed	5·20(8·04)	4·60(7·34)	0·864§
Wholly performed	1·10(2·08)	0·60(1·58)	0·552§
No. of robotic operations performed as assistant*	0·10(0·32)	0·10(0·32)	1·000§
Duration of previous laparoscopic simulation (h)*	2·00(1·25)	1·90(1·29)	0·862§
**Cholecystectomy**			
Time to complete hepatocystic triangle dissection (min)* (*n* = 5)	28·42(6·15)	38·68(0·12)	0·089§
Time to complete gallbladder liver bed dissection (min)* (*n* = 5)	18·33(4·21)	30·55(5·97)	0·025§
GRS score†	20·00 (17·75–21·75)	24·50 (21·00–28·00)	0·007¶
**Continuous suture closure**			
Time to complete closure of gastrostomy (min)*	33·25(8·42)	21·05(5·23)	0·001§
No. of continuous suture loops created†	5·00 (4·00‐6·00)	7·50 (6·75–8·25)	< 0·001¶
No. of continuous suture errors†	22·25 (20·75–25·25)	7·00 (4·75–9·63)	< 0·001¶
GRS score†	18·25 (15·88–20·63)	27·25 (22·00–28·25)	< 0·001¶
**Interrupted suture anastomosis**			
Time to complete 6 interrupted sutures (min)*	36·89(5·52)	26·59(4·48)	< 0·001§
No. of interrupted suture errors†	29·50 (23·75–31·50)	8·25 (6·38–10·13)	< 0·001¶
GRS score†	17·50 (14·50–20·13)	27·50 (22·75–29·25)	< 0·001¶

Values are

* mean(s.d.) and

† median (i.q.r.). GRS, Global Rating Scale.

‡ Fisher's exact test,

§ Student's *t* test and

¶ Mann–Whitney *U* test.

**Fig. 3:**
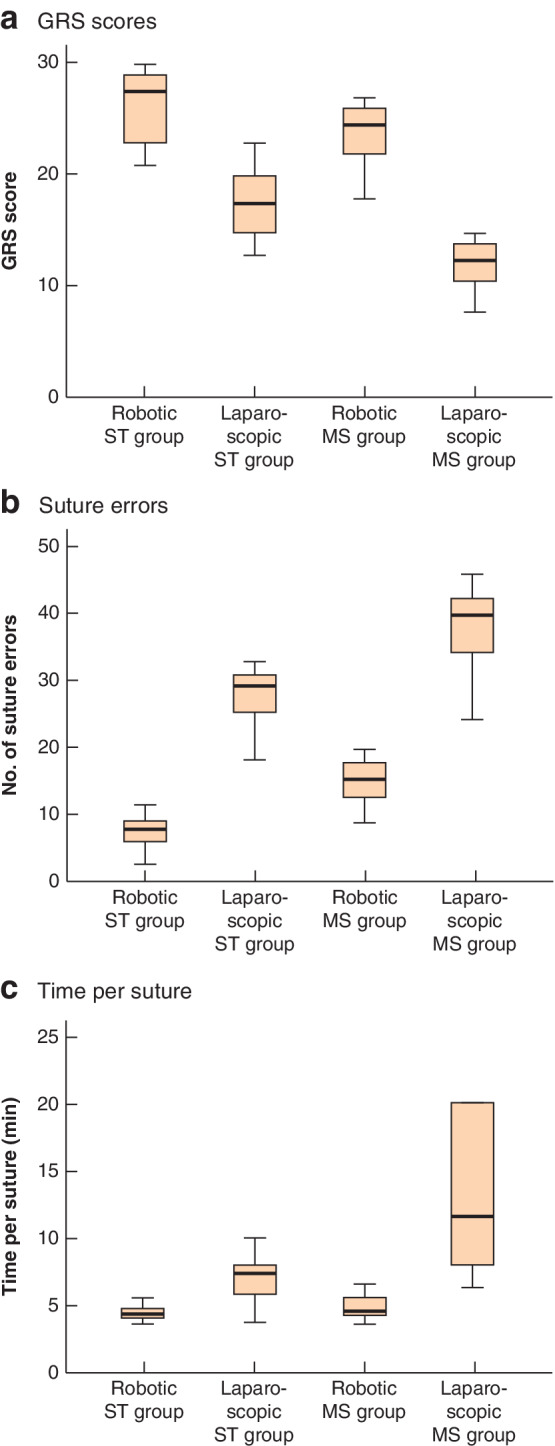
Global Rating Scale scores, suture errors and time per suture for interrupted sutures in surgical trainee and medical student groups **a** Global Rating Scale (GRS) scores, **b** number of suture errors and **c** time per suture in robotic and laparoscopic surgical trainee (ST) and medical student (MS) groups. Median values, interquartile ranges and ranges are denoted by horizontal bars, boxes and error bars respectively.

After video analysis, the mean score (GRS and number of suture errors) from both assessors was recorded for each participant for each procedure (*Table* *3* and *Fig*. *2*). For the cholecystectomy task, each participant performed either hepatocystic triangle (5 laparoscopic, 5 robotic) or liver bed (5 laparoscopic, 5 robotic) dissection. The laparoscopic group completed the liver bed dissection in a faster time than the robotic group, but had a lower GRS score for each procedure and took longer to perform the suturing tasks, with more suture errors. The median GRS score and number of suture errors for the interrupted sutures is depicted in *Fig*. *3*. When total GRS scores were combined, the median score was better after robotic compared with laparoscopic training (total GRS score: 27·00 (i.q.r. 22·25–28·33) *versus* 18·00 (16·50–19·04) respectively; *P* < 0·001).

Participants in the laparoscopic group reported a mean physical comfort level (1, extremely uncomfortable; 5, extremely comfortable) of 1·1 (range 1–3) compared with 4·6 (3–5) in the robotic group (*P* < 0·001). Participants in the laparoscopic group also reported a mean physical fatigue level (1, extremely fatigued; 5, not at all fatigued) of 2·3 (range 1‐3), *versus* 4·4 (3–5) in the robotic group (*P* = 0·002).

### Medical student group and comparison with surgical trainees

Ten medical students were randomized to the laparoscopic and ten to the robotic group. Group characteristics and the students' previous experience were comparable (*Table* *4*). No participant had any previous experience as a surgical assistant for either laparoscopic or robotic operations, and none had any robotic simulator experience.

**Table 4:** Baseline characteristics and results of medical student group

	Laparoscopic group (*n* = 10)	Robotic group (*n* = 10)	*P*
**Sex ratio (M** : **F)**	6 : 4	7 : 3	0·639‡
**Previous experience**			
Regular video gaming (h/week)*	4·30(6·82)	4·90(5·57)	0·832§
Laparoscopic simulation experience (h)*	0·60(0·94)	0·85(0·75)	0·518§
**Interrupted suture**			
No. of sutures completed†	3·50 (2·00–5·17)	8·67 (6·75–9·50)	< 0·001¶
No. of suture errors†	40·00 (37·50–50·00)	15·50 (12·25–18·50)	< 0·001¶
GRS score†	12·50 (9·25–14·50)	24·50 (21·50–26·25)	< 0·001¶

Values are

* mean(s.d.) and

† median (i.q.r.). GRS, Global Rating Scale.

‡ Fisher's exact test,

§ Student's *t* test and

¶ Mann–Whitney *U* test.

After video analysis, the mean score (GRS and number of suture errors) from both assessors was recorded for each participant (*Table* *4* and *Fig*. *2*). The median GRS score, number of suture errors and time taken to complete each suture, for both ST and MS groups, is depicted in *Fig*. *3*.

When the GRS scores of the two groups were compared for interrupted suturing, there was no difference between the robotic groups (*P* = 0·095). However, the ST group performed significantly better in laparoscopic suturing (*P* = 0·001). The median time taken for the robotic ST group to complete each suture was 4·46 (i.q.r. 3·96–4·89) min, compared with 4·62 (4·22–5·95) min for the robotic MS group (*P* = 0·382). For the laparoscopic groups, the median time taken to complete each suture was 7·30 (5·67–8·50) and 11·67 (7·88–20·00) min respectively (*P* = 0·026) (*Fig*. *3*).

### Scoring validation

Each assessor was trained in video analysis assessment and the scoring systems used by a third party. Both performed laparoscopic and robotic operations in their usual practice. To ensure that GRS and suturing error scoring was objective, interassessor reliability was assessed using the ICC. There was no significant difference between the scores for the cholecystectomy GRS (ICC 0·78), continuous suturing GRS (0·99), interrupted suturing GRS (0·99), continuous suture errors (0·98), or interrupted sutures errors (0·97). Each assessor also scored three laparoscopic and three robotic procedures at a second time point; intra‐assessor reliability did not differ using the IntraCC.

## Discussion

This study analysed the difference between acquiring surgical skills in robotic and laparoscopic operating. After receiving 6 h of either laparoscopic or robotic simulation training, 20 surgical trainees performed three operative tasks on human cadavers: an element of cholecystectomy, continuous suture closure of a gastrostomy, and interrupted sutured end‐to‐end small bowel anastomosis. The 20 medical students received 2 h of either laparoscopic or robotic simulation training followed by interrupted suture closure of a gastrostomy. The results of this randomized trial indicated that GRS scores were consistently better for the robotic groups for each task. Further, the robotic groups took less time to complete suturing tasks with fewer suturing errors, and fatigue and comfort scores were significantly better after task completion.

The GRS is a validated assessment tool for MIS operating performance^10^. It involves parameters that include respect for tissue, time and motion, instrument handling, flow of operation, depth perception, and bimanual dexterity. The robotic groups scored higher for each part of the GRS. In the medical student group of surgically naive participants, after only 2 h of simulator training the median GRS score was 24·50 for the robotic group, compared with 12·50 for the laparoscopic group. GRS scores for interrupted suturing, and the time taken to perform each suture, did not differ between the ST and MS groups, highlighting that the robotic system enabled the quick acquisition of surgical skills. With basic suturing tasks, not only did the robotic group achieve a faster completion time, participants also committed fewer errors, and more loops were created with the continuous suturing task. Again, a marked difference was seen in the MS group where, in 40 min, the robotic group completed 8·67 interrupted sutures with 15·50 errors, compared with only 3·50 sutures with 40·00 errors for the laparoscopic group.

The only advantage in the laparoscopic ST group was taking less time to complete dissection of the gallbladder from the liver bed. A slower pace in less complex operations may reflect the enhanced vision, leading novices to dissect tissue strands more meticulously, and this could account for the better GRS scores in the robotic group. There was no task‐specific simulator training for cholecystectomy, although participants in the ST group had previous exposure to laparoscopic, but not robotic, cholecystectomy, giving the laparoscopic ST group better task‐specific knowledge.

A limitation of this trial was the inability to blind the assessors to the groups. This was controlled for by having two independent assessors, as well as each assessor scoring some procedures at a second time point. In addition, all participants in the ST group had previous exposure to laparoscopic simulation and operating, but no robotic experience. In the MS group, owing to space restrictions, the laparoscopic group trained on a box trainer, whereas the robotic group trained on a virtual reality simulator. This may have contributed to the skill difference found. The MS group had only 2 h of training, compared with 6 h for the ST group. Comparisons between the groups must be taken with caution. Physical discomfort and fatigue scores were assessed using an unvalidated scoring system, and may have been lower in the laparoscopic group due to an inability to adjust the cadaveric operating table.

The present results are consistent with similar published data that analysed basic skills tasks in surgical novices and in expert laparoscopic and robotic surgeons^12^. Using box trainers, this showed a statistically significant benefit in task precision using the robotic technique, for each level of surgical experience. Those experienced in both laparoscopic and robotic surgery had fewer errors using the robotic rather than the laparoscopic trainer. A further study^13^ showed that, in a hybrid surgical simulator, time, path length and smoothness of simulated suturing was better for robotic novices than for laparoscopic novices, as well as expert laparoscopic surgeons performing better in the robotic arm. Robotic assistance also enabled medical students to suture faster and with fewer errors compared with a laparoscopic technique on a porcine fundoplication model^14^, whereas faster suturing and better dexterity skills were seen among surgeons using the robotic *versus* a laparoscopic platform^15^.

There is already evidence that the learning curve for specific operations is shorter for robotic techniques. For complex liver resections, only 16 robotic compared with 29 laparoscopic resections were required before there was improvement in the procedural difficulty index^4^. Reduced operating time is seen after fewer cases for robotic right colectomies and robotic nephrectomies compared with times for the laparoscopic technique^6^, ^8^, and in rectal cancer surgery a faster learning curve for extracorporeal and total mesorectal excision phases was seen with robotic surgery^16^.

The faster acquisition of surgical skills in robotic compared with laparoscopic surgery may have fundamental implications for future surgical training, by reducing the length of time to learn robotic skills through simulation training. Future surgeons can be trained to perform robotic operations more quickly with fewer errors than those trained as laparoscopic surgeons, emphasizing the requirement for the early introduction of robotic programmes for trainees. This should allow more patients to have access to the benefits of MIS, particularly for long and complex operations that presently deter some surgeons from performing laparoscopic procedures. Early robotic experience may accelerate minimally invasive skills acquisition, enhancing surgical training.

## Disclosure

This trial was funded by Intuitive Foundation, Sunnyvale, California, USA. The authors declare no other conflict of interest.
